# Survey of tick-borne pathogens in grazing horses in Kyrgyzstan: phylogenetic analysis, genetic diversity, and prevalence of *Theileria equi*

**DOI:** 10.3389/fvets.2024.1359974

**Published:** 2024-04-29

**Authors:** Kursat Altay, Ufuk Erol, Omer Faruk Sahin, Mehmet Can Ulucesme, Ayperi Aytmirzakizi, Munir Aktas

**Affiliations:** ^1^Department of Parasitology, Faculty of Veterinary Medicine, Sivas Cumhuriyet University, Sivas, Türkiye; ^2^Department of Parasitology, Faculty of Veterinary Medicine, Firat University, Elazig, Türkiye; ^3^Faculty of Veterinary Medicine, Kyrgyz-Turkish Manas University, Bishkek, Kyrgyzstan

**Keywords:** tick borne pathogens, Theileria equi, genotypes, horse, Kyrgyzstan

## Abstract

**Introduction:**

Tick-borne pathogens (TBP) are an important group of organisms that can affect animals and humans all over the world. Equine piroplasmosis (EP), caused by *Theileria equi* and *Babesia caballi*, is considered one of the most important tick-borne diseases and can cause significant clinical symptoms and mortality in horses. Moreover, EP plays a restrictive role in international horse traditions and transportation. Although these species can cause similar symptoms, there are different 18S rRNA genotypes of *T. equi* (five genotypes) and *B. caballi* (three genotypes). Besides piroplasma species, *Anaplasma* and hemotropic mycoplasmas (HM) are known as other important tick-borne pathogens reported in horses.

**Methods:**

In this study, we investigated the presence, prevalence, genetic diversity, and phylogenetic analyses of TBPs using PCRs and DNA sequencing in grazing horses in Kyrgyzstan. For these purposes, a total of 311 blood samples were collected from Chuy, Issyk-Kul, Naryn, Osh, Talas, and Jalal-Abad.

**Results:**

DNA amplification of TBP revealed that 23 (7.40%) out of 311 samples were found to be positive for *T. equi*. However, *B. caballi*, HM, *A. phagocytophilum*, and *A. capra* were not detected in this study. The infection rate of *T. equi* was higher in males (8.11%) than in females (6.35%) (*p*=0.2880) and in those older than 5 years (9.02%) than in the 1-4 age group (6.35%) (*p*=0.1950). Phylogenetic analysis of 18S *rRNA* revealed that A and E genotypes of *T. equi* have circulated in grazing horses in Kyrgyzstan.

**Discussion:**

Information about the genetic diversity of *T. equi* is important for understanding the population dynamics of the species and developing effective control strategies against this pathogen. This is the first molecular investigation of *A. capra* in horses in Kyrgyzstan. Although this pathogen has been detected in different hosts in Kyrgyzstan, it was not detected in this study. However, considering the wide host spectrum of *A. capra*, it is thought that more large-scale studies are needed to understand the effect of horses on the epidemiology of this pathogen.

## 1 Introduction

Tick-borne pathogens (TBP) are an important group of organisms that can affect animal and human health all over the World (1). Equine piroplasmosis (EP), equine anaplasmosis (formerly Equine Granulocytic Ehrlichiosis), and hemotropic mycoplasmas (HM) are the main TBPs of horses (2–4). EP is caused by *Theileria equi* (formerly *Babesia equi*), *Babesia caballi*, and the recently described *T. haneyi*. The disease is observed in horses, mules, donkeys, and zebras (2, 5, 6). EP is a global disease and a few countries such as Australia, Canada, Great Britain, Ireland, Japan, and New Zealand have EP-free status (5, 6). Ixodid ticks such as *Dermacentor*, *Hyalomma*, and *Rhipicephalus* are the biological vectors of EP, and it is mainly transmitted by these vectors. In addition, it has been reported that it is transmitted through contaminated needles, surgical operations, blood transfusion, and transplacental (2, 6, 7). EP exhibits a clinical course ranging from subclinical to acute. *Theileria equi* causes more severe clinical disease than *B. caballi.* The clinical signs of EP are fever, anemia, jaundice, and hemoglobinuria (6, 8). The mortality can reach up to 50% in *T. equi* infections, while 10% in *B. caballi* infections (6, 8). Horses that recover from the disease remain carriers for a long time. While in *T. equi* the carrier period lasts lifelong, in *B. caballi* this period is about 4 years (9). EP causes significant economic losses in the equine industry worldwide and even has a restrictive effect on international horse movements (2, 6).

Equine anaplasmosis (formerly Equine Granulocytic Ehrlichiosis) is a tick-borne disease of horses caused by *Anaplasma phagocytophilum* (formerly *Ehrlichia equi*). It has a very wide host spectrum and infects horses, cattle, sheep, goats dogs, cats, and humans (10). The clinical signs occur mostly in adult horses, which include fever, anorexia, depression, reluctance to move, limb edema, petechiation, and icterus (10–12). *Anaplasma capra* is discovered for the first time in goats from China in 2012 (13). In the short time so far, *A. capra* has been detected in many different countries including Kyrgyzstan from three continents (Africa, Asia, and Europe) (14–26). The studies show that the *A. capra* can infect a wide range of host groups including sheep, goats, cattle, water buffaloes, dogs, wild animals (e.g., deer, takin, Persian onegar, muntjac, serow), and humans (13–18, 20–25). Additionally, it was detected in ticks (19). There is no information regarding the presence of *A. capra* in horses. However, *A. capra* has been detected in wild onegars (*Equus hemionus onager*) in Iran (24).

Hemotropic mycoplasmas or hemoplasmas refers to the infection caused by small, cell wall-less Gram-negative bacteria (*Mycoplasma* spp., class *Mollicutes*) that attach to the surface of red blood cells, formerly known as eperythrozoonosis and haemobartonellosis. These species infect many animal species (26). The diseases may be transmitted by arthropod vectors such as ticks, lice, flies, and mosquitoes. Additionally, the disease can be transmitted through blood transfusion, contaminated needles or surgical equipment, and vertically (26). Information about HM in horses is scarce. The disease was first detected in horses by microscopic examination of blood smears in Nigeria in 1978 (4). In 2010, the first molecular diagnosis of the disease in horses was made and the species detected in horses from Germany were determined closely related to *Mycoplasma haemofelis* and *Candidatus Mycoplasma haemobos* (27). Apart from Germany (27) studies on molecular basis determination of HM in horses were conducted in Iran (28), Nigeria (29), and Brazil (30).

To date, no record of equine-TBPs has been found in the literature in Kyrgyzstan. Recently, it has been reported first molecular presence and prevalence of the important TBPs such as canine and bovine hemotropic mycoplasma species (31, 32), *B. vogeli, B. vulpes* in dogs (33), *A. centrale, A. capra*, *A. phagocytophilum*-like 1, *B. major, T. annulata,* and *T. orientalis* in cattle (14, 15, 34, 35), *A. ovis, A. capra*, *and A. phagocytophilum*-like 1 in sheep (36) in Kyrgyzstan. In this study, we aimed to survey of TBPs (EP, HM, *Anaplasma phagocytophilum,* and *Anaplasma capra*) in grazing horses in Kyrgyzstan and to detect the genetic diversity of the pathogens.

## 2 Materials and methods

### 2.1 Study area, collection of blood samples, and DNA extraction

**Figure 1:**
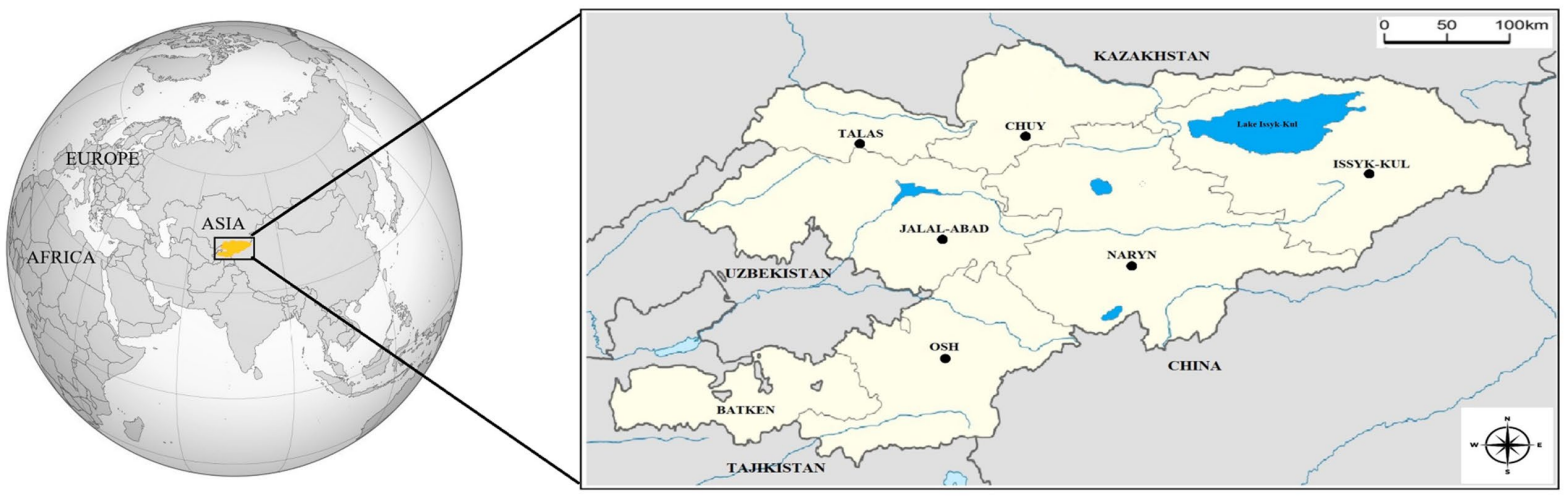
Location of Kyrgyzstan on the world map (Yellow). Neighbors and geographical regions of Kyrgyzstan. Sampling regions were shown with circles.

The Republic of Kyrgyzstan is a Central Asia country bordered by Uzbekistan to the west, China to the east and southeast, Tajikistan to the south, and Kazakhstan to the north. The country is generally covered with high mountains and has a continental climate, with hot summers and cold winters. While the summers in low-altitude settlements can get quite hot, the high mountains remain colder even in the hottest months. It consists of seven geographical regions (Talas, Naryn, Batken, Issyk-Kul, Chuy, Osh, and Jalal-Abad) (Figure 1) (37).

The blood samples were collected from 311 grazing horses from 35 flocks in 21 settlements in 6 geographical regions (Chuy, Osh, Naryn, Talas, Jalal-Abad, and Issyk-Kul) of Kyrgyzstan (Figure 1) between March 2022 and July 2023. Horse flocks in the settlements selected for the study and the animals from which blood was obtained in the flocks were randomly selected. The age and gender of the animals were recorded (Table 1). There were not any clinical signs on horses at first examination and they were recorded as healthy.

**Table 1:** The numbers of blood samples by location, age and gender.

Locations	Age (year)		Gender		Total
	1–4	5≤	Male	Female	
Chuy	102	86	124	64	188
Issyk-Kul	30	6	19	17	36
Naryn	20	10	12	18	30
Osh	15	7	12	10	22
Talas	13	7	10	10	20
Jalal-Abad	9	6	8	7	15
Total	189	122	185	126	311

The total genomic DNAs were obtained from blood samples using a commercial DNA isolation kit (GeneAll Exgene^™^ Clinic SV kit, 108–152, GeneAll^®^, Seoul, South Korea). These gDNA samples were sored −20°C until use.

### 2.2 Survey of equine piroplasmosis, hemotropic mycoplasmas, *Anaplasma phagocytophilum*, and *Anaplasma capra* by polymerase chain reaction

The four different polymerase chain reaction (PCR) were used for the survey of equine piroplasmosis (EP), hemotropic mycoplasmas (HM), *Anaplasma phagocytophilum,* and *Anaplasma capra* DNAs in the study. A nested PCR was carried out for the amplification of *Theileria* spp. and *Babesia* spp. *18S rRNA* gene. First, 1,600 bp of the *18S rRNA* gene of *Theileria* spp. and *Babesia* spp. was amplified by PCR from the samples with Nbab-1F and Nbab-1R primers (38). In nested PCR using these PCR products as templates, ~500 bp fragments of the same gene was amplified with BJ1 and BN2 primers (39). To survey of HM species in the samples, the primers amplifying 192 bp fragments of *16S rRNA* gene were used in the PCR (40). To survey of *A. phagocytophilum* in the samples SSAP2f and SSAP2r primers amplifying 641/642 bp fragments of *16S rRNA* gene were used in the PCR (41). *Anaplasma capra* was researched by a nested PCR in the samples. First, 1,031 bp fragments of the *gltA* gene (20) and then 594 bp fragments of the same gene (19) were amplified by PCR. The detailed information about PCRs and the primers is presented in Table 2.

**Table 2:** The characteristics of the primers used in this study.

Species	Primer name	Primer sequence (5′-3′)	Target gene	Amplicon size (bp)	Annealing temperature (°C)	References
*EP* (*Theileria* spp. and *Babesia* spp.)	*Nbab_1F	AAGCCATGCATGTCTAAGTATAAGCTTTT	*18S rRNA*	1,600	56	Oosthuizen et al. (38)
	*Nbab_1R	CTTCTCCTTCCTTTAAGTGATAAGGTTCAC				
	**BJ1	GTCTTGTAATTGGAATGATGG		~500	56	Casati et al. (39)
	**BN2	TAGTTTATGGTTAGGACTACG				
HM	Forwad	ACGAAAGTCTGATGGAGCAATA	*16S rRNA*	192	52	Jensen et al. (40)
	Reverse	ACGCCCAATAAATCCG(A/G)ATAAT				
*A. phagocytophilum*	SSAP2f	GCTGAATGTGGGGATAATTTAT	*16S rRNA*	641–642	54	Kawahara et al. (41)
	SSAP2r	ATGGCTGCTTCCTTTCGGTTA				
*A.capra*	Outer-f	GCGATTTTAGAGTGYGGAGATTG	*gltA*	1,031	50	Li et al. (20)
	Outer-r	TACAATACCGGAGTAAAAGTCAA				
	Inner-f	TCATCTCCTGTTGCACGGTGCCC		594	57	Yang et al. (19)
	Inner-r	CTCTGAATGAACATGCCCACCCT				

EP, equine prioplasmosis, HM, hemotropic mycoplasmas, *: outer primers, **: inner primers.

The PCR assays were performed as described before (19, 20, 38–41), and the genomic DNA of *B. vogeli* (Accession number: OR116199) (33), *A. phagocytophilum* (Accession number: OP828919) (42), *A. capra* (Accession number: ON783818) (22), and *M. wenyonii* (Accession number: OM468183) (43) were used as the positive controls, and DNase-RNase-free sterile water (Cat No.: 129114, Qiagen^®^, Germany) was used as the negative control in the PCRs.

PCR products were loaded on 1.5% agarose gel containing ethidium bromide and visualized under a UV transilluminator. The DNA extraction, PCR, and gel electrophoresis were performed in separate compartments of the laboratory to minimize the risk of contamination.

### 2.3 Sequencing and phylogenetic analysis

All positive samples obtained in the study were sequenced using the primers listed in Table 1.

The amplicons were purified from agarose gel using a commercial gel extraction kit (PCR Clean-Up & Gel Extraction Kit, GeneDireX^®^, Cat.No.: NA006-0300). The purified products were sent to bidirectionally sequence (ABI 3730XL analyzer, Applied Biosystems, Foster City, CA). The BigDye Terminator v3.1 Cycle Sequencing Kit was used in the reactions (Applied Biosystems, Foster City, CA).

The obtained sequences were aligned with each other with the MUSCLE algorithm of MEGA-11 software (44), and consensus sequences were constructed. Accession numbers were obtained by submitting them to GenBank. Phylogenetic analyses of gene sequences identified in this study were performed and genotypes of the *T. equi* were determined.

**Figure 2:**
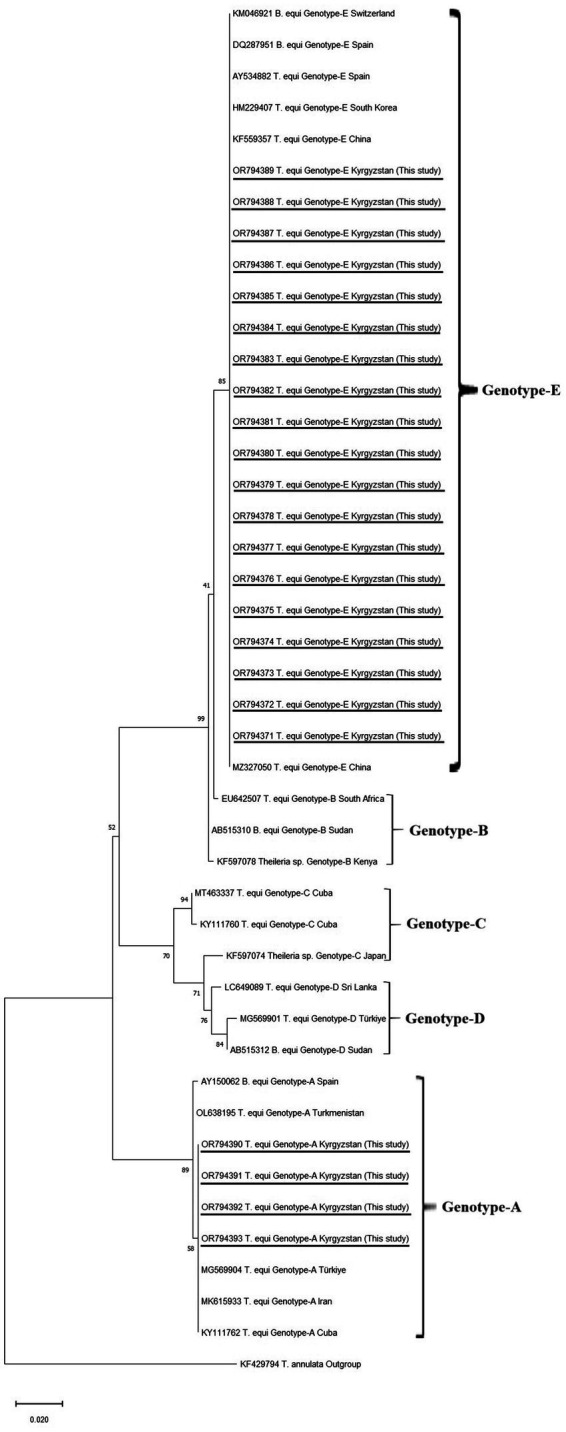
Phylogenetic trees according to *18S rRNA* sequences of *Theileria equi* genotypes. The figure was created using the ML method. Bootstrap values were performed with 1,000 replicates. The evolutionary history was performed by using the ML method TN93 + G model were used of *T. equi* genotypes (45). Evolutionary analyses were conducted in MEGA-11 (44). *T. annulata* was used as an outgroup.

The best-fit model for maximum likelihood was determined as the TN93 + G parameter model (45) using the Find Best-Fit Substitution Model in MEGA-11 (44). The phylogenetic trees were created using maximum likelihood analysis in Mega 11 (44) (Figure 2).

### 2.4 Statistical analysis

The chi-square test (χ^2^) was used to determine the differences among various parameters. *p* < 0.05 was accepted to be statistically significant.

### 2.5 Ethics statement

The ethical permission was granted from the Kyrgyz-Turkish Manas University Animal Experiments Local Ethics Committee with number 2023/12. The owners of the horses also gave their oral permission to collect the blood samples from their animals.

## 3 Results

### 3.1 The results of PCRs

A total of 311 horse blood samples were analyzed by equine piroplasmosis (EP), hemotropic mycoplasmas (HM), *Anaplasma phagocytophilum,* and *Anaplasma capra* specific-PCRs. As a result of PCRs, positivity was detected in 23 samples only with EP specific PCR, while no positivity was detected in the other three PCRs.

The 23 positive samples were identified as *T. equi* by sequencing (section of sequencing of *18S rRNA* gene and phylogenetic analysis). Accordingly, PCR results based on sequence analyses are given in Table 3. The total prevalence of *T. equi* was determined as 7.40% (23/311). This rate was found to be 8.11% in males (15/185) and 6.35% in females (8/126) (*p* = 0.2880). It was determined as 6.35% (12/189) in the 1–4 age group and 9.02% (11/122) in those aged 5 and older (*p* = 0.1950) (Table 3).

**Table 3:** *Theileria equi* positive samples by location, age and gender.

Locations	Age		Gender		Total
	1–4	5≤	Male	Female	
	% (+/*n*)	% (+/*n*)	% (+/*n*)	% (+/*n*)	% (+/*n*)
Chuy	8.82 (9/102)	11.63 (10/86)	10.48 (13/124)	9.38 (6/64)	10.11 (19/188)
Issyk-Kul	6.67 (2/30)	16.67 (1/6)	10.53 (2/19)	5.88 (1/17)	8.33 (3/36)
Naryn	5 (1/20)	0 (0/10)	0 (0/12)	5.56 (1/18)	3.33 (1/30)
Osh	0 (0/15)	0 (0/7)	0 (0/12)	0 (0/10)	0 (0/22)
Talas	0 (0/13)	0 (0/7)	0 (0/10)	0 (0/10)	0 (0/20)
Jalal-Abad	0 (0/9)	0 (0/6)	0 (0/8)	0 (0/7)	0 (0/15)
Total	6.35 (12/189)	9.02 (11/122)	8.11 (15/185)	6.35 (8/126)	7.40 (23/311)

%: percentage, +: number of positive samples, n: number of samples.

### 3.2 Sequencing of *18S rRNA* gene and phylogenetic analysis

Equine piroplasmos (EP) was detected in 23 samples by PCR. The partial sequence analyses of *18S rRNA* revealed that 19 positive samples were matched with the *T. equi* E genotype, whereas four samples were *T. equi* A genotypes. The consensus sequences were uploaded to the GenBank under accession numbers *T. equi* E genotypes: OR794371-OR794389, *T. equi* A genotypes: OR794390-OR794393.

The sequences of *T. equi* E genotypes identified in this study were 100% identical to each other. High nucleotide similarities (99.78–100%) were seen between *T. equi* E genotypes obtained in this study and *T. equi* E genotype uploaded to the GenBank in different parts of the world. Furthermore, 100% nucleotide identities were present between our isolate and *T. equi* isolates identified from Ukraine (KP868757), China (OQ692565 and MZ327270), Russia (OM475525), Austria (MW446331), and Portugal (MT767169).

*Theileria equi* A genotypes obtained in this study had 100% nucleotide identities with each other. The 99.35–100% nucleotide similarities were determined between our sequence and *T. equi* A genotypes present in the GenBank deposited from various countries. The *T. equi* A genotype sequences obtained in this study showed 100% nucleotide similarities to those of *T. equi* A genotypes identified in Türkiye (MG569905), France (MK732476, MF510478), Portugal (MT767167), Israel (MK392060, MK063843), Saudi Arabia (LC431545, KJ801931), Egypt (MN625898), Chile (MT463613), Brazil (MG052917, KY952237), Cuba (KY111762), and United States (CP099438 and JX177673).

The phylogenetic tree showed that our *T. equi* Genotype-E and Genotype-A clustered with Genotype-E and Genotype-A identified from different countries, respectively.

## 4 Discussion

The factors such as global warming, increased use of rural areas, animal movements, and the contribution of migratory birds increase the spread and importance of TBPs (1). Knowing basic epidemiological information plays a critical role in the development and implementation of effective and widespread control methods for these diseases. Obtaining the most epidemiological data can undoubtedly be achieved through studies using methods with high specificity and sensitivity in different regions of the world. Equine piroplasmosis, an important TBP of horses, can be diagnosed by microscopic, serological, and molecular techniques (6). Microscopic examination of stained blood smears has the disadvantage of low sensitivity, especially in chronic infections with low parasitemia. The complement fixation technique, the indirect fluorescence antibody test, and the competitive inhibition enzyme-linked immunosorbent assay are used to detect parasite-specific antibodies (6). The most important disadvantage of serological methods is that they do not distinguish between current and previous infections (8). Molecular diagnostic techniques, especially PCR, stand out with their superiority in both specificity and sensitivity in the diagnosis of EP. In recent years, these methods have been widely used in epidemiological studies (46–50). Although the presence of different TBPs in cattle, sheep, and dogs (HM species, *B. vogeli, B. vulpes, A. centrale, A. capra*, *A. phagocytophilum-*like 1, *A. ovis*, *B. major, T. annulata,* and *T. orientalis*) has been previously reported in Kyrgyzstan (14, 15, 31–36), this study is the first molecular study investigating TBPs in horses in the country.

In recent studies, it has been shown that EP agents are divided into five genotypes (A, B, C, D, and E) based on the *18S rRNA* gene (46, 51–53). Moreover, it has been reported that the *T. equi*-C genotype includes the species described as *T. haneyi* (5). In 2001, it was reported that *T. equi* has two genotypes (Florida (United States) and Pelotas (Southern Brazil) genotypes) based on the *EMA-1* gene (54). The *18S rRNA* gene has advantages in genotyping studies due to the fact that it has multiple copies in the genome and the hypervariable region is also located here (55, 56). It has been used extensively in studies on this subject. Firstly, *B. equi* Spain 1 and *B. equi* Spain 2 were reported in 2003, based on the *18S rRNA* gene (57). In another conducted in Spain in 2004, *B. equi* Spain 1 and *B. equi* Spain 2 isolates were located in the same cluster, while *T. equi*-like was located in a different cluster, thus supporting the existence of two genotypes based on two *18S rRNA* genes (58). In a more comprehensive study conducted in South Africa in 2009, a third genotype was detected, and the genotypes up to that time were named genotypes A, B, and C (59). According to the study, *B. equi* Spain-1 and *B. equi* Spain-2 isolates detected from horses and *T. equi* isolate detected from dogs in Spain (57) were collected under genotype A; *T. equi*-like isolates detected from horses in Spain and (58), and from zebra in South Africa (59) were collected genotype B, and *T. equi* isolates detected from horses in South Africa (59) were collected under genotype C (59). Following these, the presence of genotype D in horses in Sudan was revealed in 2010 (60), and genotype E was revealed in 2012 based on previous South Korean-*T. equi* sequences (61). In this study, 23 samples were sequenced. As a result of phylogenetic analysis based on *18S rRNA* gene DNA sequences, we identified two genetically distinct *T. equi* genotypes (A—4 samples and E—19 samples) in grazing horses from Kyrgyzstan. This is the first study that reports of *T. equi* and its genotypes in Kyrgyzstan. Our results confirm that different genotypes of *T. equi* coexist within the same population and the heterogeneous nature of this species, in agreement with previous studies (46, 51–53, 62). However, there is still a need for molecular surveys to determine the geographical distribution of the genotypes and their impact on the course of clinical infections, as well as studies on differences in vectors.

The PCR-positivity for *T. equi* was found to be 7.40% (23/311). While it is close to the rate previously reported in horses in Türkiye (8.8%) (63), it is generally lower than the positivity rates reported in many parts of the world such as 19.7% in Türkiye (49), 38.9% in China (50), 44.5% in Spain (64), 61.9% in Gambia (65), 70.3% in Italy (66), 73.0% in Cuba (47). The differences among *T. equi* positivity rates may be related to sample numbers, the method used in the studies, age groups, animal management system, ecological factors, tick species in the region, and other risk factors.

The age of horses may be considered a risk factor for *T. equi* infections due to longer tick exposure. Additionally, the fact that *T. equi* is a lifelong carrier in horses may increase the percentage of positive older horses (6, 67). Bartolomé del Pino et al. (66) reported *T. equi*-PCR-positivity significantly decreases with age in Itay. On the other hand, Rueg et al. (68) reported that *T. equi* positivity increased with age in Mongolia. Unlike both cases, it has been reported that the *T. equi*-PCR-positivity rate has no connection with age in Türkiye (69). These different results may be due to multivariate factors related to long-term parasite host circulation processes, which need to be explained on the basis of host–parasite interaction, and the sensitivity of the methods used may also affect this situation. Although the prevalence of *T. equi* was high in horses older than 5 years of age in this study, this difference was found to be statistically insignificant (*p* = 0.1950). On the other hand, all animals examined in this study appear healthy. When all the data are evaluated together, it may contribute to the understanding of the long-term carrier status of horses. The decrease in prevalence with age in *B. caballi* is attributed to the clearance of the agents in 4 years and the loss of antibodies in the following period (66). This may be related to the overall low prevalence of *B. caballi* and the fact that it was not detected in this study.

While there are studies showing that gender is related to *T. equi* positivity (64, 70, 71), there are also studies showing that gender is not a risk factor (72, 73). In our study, *T. equi* prevalence by gender was found to be statistically insignificant (*p* = 0.2880). It is thought that this situation may be due to the husbandry techniques.

Equine anaplasmosis is mainly common in regions where *Ixodes* species occur, especially in northern America, and is also seen in Europe, Africa, and Southern America (3, 12, 74). It has been determined that the prevalence of *A. phagocytophilum* in Germany peaked in relation to the activity of *I. ricinus* (75). *Anaplasma phagocytophilum-*PCR positivity in horses was detected as 13% in the humid region of Tunisia (76), 8% in Italy (77), 4.3% in Pakistan (77), and 15% in the island of Sardinia (74). Although *A. capra* was detected in 2012 (13), it was included in this study because it was found in a very large host group and was found in wild onegar in Iranian (24), a species relatively close to horses. There has been no previous study investigating *A. capra* in horses. *Anaplasma capra* and *A. phagocytophilum* could not be detected in horses in our study. Unlike the above regions, Kyrgyzstan is a very mountainous country with no sea borders. The continental climate of the country and the related tick fauna may directly affect the presence and prevalence of TBPs. In our study, the animals whose blood was collected appeared healthy and no ticks were found. However, more comprehensive studies including tick species in the country are needed.

There is a paucity of information on the prevalence and distribution of hemotropic mycoplasma species in horses. Molecular-based studies have shown that the hemoplasma species detected in horses are species associated with different animal species (27–30). In these studies, *Canditus M. haemobos* and *M. haemofelis*, which are related to cattle and cats, respectively, in Germany (27), *M. ovis* which is mostly related to sheep in Brazil (30), *Mycoplasma ovis*-like and *Candidatu*s *M. haemocervae* were detected in horses from Iran and Nigeria, respectively (28, 29). Besides this, in three different studies investigating TBPs in horses in Brazil, the presence of *T. equi* and *B. caballi* was reported, but hemotropic mycoplasma could not be detected in the horses (78–80). Additionally, some researchers have stated that hemoplasma infections may be accidental or an uncommon disease in horses (78). In this study, hemoplasma species were not detected in horses, and this may be related to the absence of hemoplasma species in other animals in the sampling areas.

## 5 Conclusion

The global importance of TBPs is increasing among hosts, and equine piroplasmosis is the most important TBD in horses. Although different genotypes of *B. caballi* and *T. equi* have been detected, the clinical relationship, geographical distribution, and vectors of these genotypes need to be revealed. This study revealed the existence of A and E genotypes of *T. equi* in grazing horses from Kyrgyzstan, and this information helps to understand the epidemiology of these *T. equi* genotypes. In this study, *A. phagocytophilum, A. capra,* and HM were detected in horses. *A. capra*, a relatively novel species, is thought to have a global distribution, and large-scale studies are still needed to understand the prevalence, distribution, and pathogenesis of this pathogen in horses.

## Data availability statement

The datasets presented in this study can be found in online repositories. The names of the repository/repositories and accession number(s) can be found below: https://www.ncbi.nlm.nih.gov/genbank/, OR794390; https://www.ncbi.nlm.nih.gov/genbank/, OR794391; https://www.ncbi.nlm.nih.gov/genbank/, OR794392; https://www.ncbi.nlm.nih.gov/genbank/, OR794393.

## Ethics statement

The animal studies were approved by Kyrgyzs-Turkish Manas Unıversity Animal Experiments Local Ethics Committee with decision number 2023/12. The studies were conducted in accordance with the local legislation and institutional requirements. Written informed consent was obtained from the owners for the participation of their animals in this study.

## Author contributions

KA: Conceptualization, Data curation, Formal analysis, Investigation, Methodology, Project administration, Resources, Software, Supervision, Validation, Visualization, Writing – original draft, Writing – review & editing. UE: Conceptualization, Data curation, Formal analysis, Investigation, Methodology, Validation, Writing – review & editing. OS: Data curation, Formal analysis, Investigation, Methodology, Writing – review & editing. MU: Data curation, Formal analysis, Investigation, Writing – review & editing. AA: Data curation, Formal analysis, Investigation, Methodology, Writing – review & editing. MA: Conceptualization, Data curation, Formal analysis, Investigation, Methodology, Resources, Writing – original draft, Writing – review & editing.
